# Turnaround Time of the Hematology Results of Cancer Patients During the COVID-19 Pandemic: An Opportunity to Initiate a Quality Improvement Process

**DOI:** 10.7759/cureus.61149

**Published:** 2024-05-27

**Authors:** Asmae El Assil, Souad Benkirane, Yasmine El Kettani, Ali Cherif Chefchaouni, Hassane Mamad, Younes Rahali, Azlarab Masrar

**Affiliations:** 1 Central Laboratory Hematology, Ibn Sina University Hospital Center, Rabat, MAR; 2 Faculty of Medicine and Pharmacy, Mohammed V University, Rabat, MAR; 3 National Institute of Oncology, Ibn Sina University Hospital Center, Rabat, MAR

**Keywords:** covid-19 pandemic, oncology, neutropenia, hematology, turnaround time

## Abstract

Introduction: Turnaround time (TAT) is a crucial clinical parameter that reflects the performance of a laboratory especially in the context of oncology and the COVID-19 pandemic. Based on the Lean Six Sigma methodology, we performed a retrospective analysis of the TAT of the complete blood count (CBC) of cancer patients with the aim of reducing this delay in the future.

Materials and methods: Over one month of the COVID-19 pandemic, a retrospective evaluative audit was carried out on the TAT of the CBC in an oncology department. The root causes of failures of the overall analysis process were detected. The initiation of an improvement approach was implemented through the creation of an improvement flowchart and a new request form. The hospital information system (HIS) data were exported to Microsoft Excel® (Microsoft Corporation, Redmond, Washington, United States). Using the collected data, the mean, standard deviation, median, and interquartile range were calculated using IBM SPSS Statistics for Windows, Version 23, (Released 2015; IBM Corp., Armonk, New York, United States). All time intervals were expressed in minutes.

Results: Among 263 intra-laboratory TATs analyzed, the median intra-lab TAT was 56 minutes (interquartile range (IQR): 36-80 minutes). A total of 82% of the analyses were performed in less than 90 minutes with a predominance of the interval 30-59 at 42.9%. The main causes of failures were essentially the lack of time stamping of the samples as well as the lack of real-time communication between the biologists and the clinicians. The proposed improvement model is currently being approved by all practitioners whose main items are as follows: At the clinical department level, distinguish the request forms but also the labels of the samples of the oncology hospital by a particular color, indication of clinical signs and sampling time on the request forms and on the HIS. At the laboratory level, create a specific chain for oncology department samples, alarm notification on the HIS, and rapid telecommunication of results for vital situations.

Conclusion: The intra-lab TAT of our study is biologically acceptable. Because our work is limited by the phases outside the control of the laboratory, it should lead to a continuous improvement project.

## Introduction

The COVID-19 pandemic represents the greatest public health challenge of the current generation. It is transmitted by an infectious disease caused by the severe acute respiratory syndrome coronavirus 2 (SARS-CoV-2) [1,2]. Compared to the general population, cancer patients are at increased risk of infection and complications during COVID-19 [3-5].

Neutropenia is the most frequent side effect of chemotherapy. It is associated with the risk of serious life-threatening infections as well as a therapeutic arsenal delay due to the restriction of chemotherapy doses [6]. When cancer involvement is associated with COVID-19 infection, the mortality rate is significantly higher in neutropenic patients [7-9]. According to the United Kingdom National Institute for Health and Care Excellence, the symptoms of COVID-19 and neutropenia may be difficult to differentiate at the initial consultation [10]. Increased attention to the management of febrile neutropenia is recommended to minimize the risk of infection in cancer patients and reduce the need for hospital visits [7,8].

The laboratory has a crucial role in providing timely and reliable results for proper clinical decision-making. In order to achieve this objective, the laboratory can adopt a quality approach that consists of improving its results' turnaround time (TAT) [11]. The TAT is a crucial parameter in the clinic that reflects the performance of a laboratory and the efficiency of all the analysis processes from the pre-analytical phase to the post-analytical phase [12,13]. Currently, the Lean Six Sigma (LSS) method is the most adopted methodology by several laboratories in Europe and America and their impact has been well illustrated [11,14-16]. LSS is the combination of two quality methods, which aims to identify and eliminate failures and improve the quality of a process. It is based on a structured approach to problem-solving abbreviated as DMAIC, which expands to Define, Measure, Analyze, Improve, and Control [11,17,18].

Based on the LSS methodology, the aim of our work was initially to carry out a retrospective analysis of the TAT of the complete blood count (CBC), essentially the absolute neutrophil count (ANC) of cancer patients. Following this, we set out to identify the root causes of failures in the total analysis process and finished by initiating a quality improvement approach.

## Materials and methods

Study design

This study is part of a clinical-biological quality approach between the central hematology laboratory of the Ibn Sina University Hospital Center (UHC), Rabat, Morocco, and the National Institute of Oncology, Rabat, Morocco. It was a retrospective observational study over a period of one month from June 1 to July 1, 2022 (when Morocco noted a high peak of cases tested COVID-19 positive), in order to improve the TAT for the results of the CBC (ANC) of cancer patients referred to the Oncology hospital.

Pre-intervention

The central hematology laboratory treats all requests from the various services of 10 hospitals detached from the UHC, including the oncology hospital. Every day our laboratory processes an average of 700 analyses per day, which corresponds to 21,000 per month. The laboratory operates 24 hours a day in three shifts: in the morning (from 8 AM to 3 PM), in the afternoon (from 3 PM to 8 PM), and the night shift (from 8 PM to 8 AM).

For inpatients, samples are collected, barcoded, and sent to the central laboratory receiving unit via an ambulance driver. Appropriate and adequate samples are accepted, recorded in the electronic hospital information system (HIS), and analyzed. Test results are validated, interpreted, and released via the HIS.

Data collection and statistical analysis

Data items which included sample registration, acquisition to HIS, and authorization of results were extracted from the HIS onto Microsoft Excel® (Microsoft Corporation, Redmond, Washington, United States). Thereafter, analysis was done using IBM SPSS Statistics for Windows, Version 23, (Released 2015; IBM Corp., Armonk, New York, United States). Descriptive statistics, including median, percentages, and interquartile range (IQR) were calculated. Intra-lab TAT was expressed as median with IQR. All time intervals were presented in minutes. The establishment of the control chart of the first phase was also carried out in Excel software (Microsoft Corporation, Redmond, Washington, United States).

Intervention: the LSS methodology

We initiated with the application of the LSS methodology and finished by modeling the future state. The main tools of this methodology are the five phases of DMAIC, which expands to define, measure, analyze, improve, and control.

A. Define the Problem

Neutropenia in cancer patients undergoing chemotherapy is a severe hematologic toxicity and is associated with the risk of life-threatening infections, especially if accompanied by COVID-19 infection. Any delay in the laboratory's reporting of the CBC results (ANC) negatively impacts the timely management of these patients. Based on this observation, after brainstorming conducted by our multidisciplinary team, we decided to initiate a quality approach with the aim of satisfying the patient and all practitioners.

B. Measure

Due to the lack of time stamping of the pre-analytical phase from patient sampling to the reception of the samples in the laboratory, it is possible to determine only the timelines related to the analytical and post-analytical phases.

On 263 processed CBC reports, we determined two intervals: Interval 1: from registration to acquisition on HIS, and Interval 2: from acquisition to validation on HIS in addition to the delay of intra-laboratory results (TAT-intra lab), which is defined by the time between specimen registration in the HIS and the release of the validated biological results.

C. Analyze

We used a control chart to further analyze the temporal evolution of intra-laboratory TAT. A control chart was constructed by Excel software (Microsoft Corporation, Redmond, Washington, United States). To build the control chart, we used the TAT of the results obtained respecting the following limits: the central limit (CL), the upper control limit (UCL), and the lower control limit (LCL). These limits are calculated according to validated mathematical formulas in force from the process data (Association Française de Normalisation (AFNOR) standards) [19]. The chart used is the average chart or X-bar chart, which allows the variation between the averages of the subgroups of the process indicator to be followed chronologically.

To explain the causes of the delay of the intra-lab TAT, the constraints responsible for the lack of calculation of the global TAT during the global analysis process, as well as the reasons for the dissatisfaction of all practitioners, we used an Ishikawa diagram. 

D. Improve

On the basis of the analysis, corrective actions presented as a flowchart were proposed to bring in improvement for a later evaluation. We tried to limit the human factor as much as possible by using reliable schedules from the HIS. Nevertheless, the purpose of using a new request form was to expose ourselves to measurement bias (see Appendices). An indefinite period was left for their full approval and eventual implementation.

E. Control

The evaluation phase, which was intended to monitor the evolution and deduce the impact of the measures implemented, is modeled in this work. It will be the subject of another study.

## Results

Measurement results: analytical and post-analytical phases

The majority of the analyses (82%) were performed in less than 90 minutes with a predominance of the interval (30-59 minutes) at 42.9%. In contrast, only 12.6% were obtained in less than 30 minutes. Our results showed that 86% of the assessments were processed in less than 60 minutes for interval 1 and 66.6% in less than 30 minutes for interval 2 (Table 1). The intra-lab median was 56 minutes (IQR: 36-80 minutes). The median for interval 1 was greater than that for interval 2, 29 minutes (IQR: 20-40 minutes) and 20 minutes (IQR: 09-35 minutes), respectively (Table 2). The different TATs of the results obtained are presented in Table 1 and Table 2.

**Table 1:** Contribution of all turnaround times of results in minutes from the analytical to the post-analytical phase HIS: hospital information system; TAT: turnaround time

Time (minutes)	Interval 1: registration acquisition on HIS, n=263		Interval 2: acquisition validation on HIS, n=263		Intra-lab TAT, n=263	
	n	%	n	%	n	%
<30	135	51.72	174	66.66	33	12.64
30-59	93	35.63	65	24.90	112	42.91
60-89	14	5.36	18	6.89	69	26.43
90-119	10	3.83	1	0.38	24	9.19
120-149	4	1.53	2	0.76	8	3.06
>150	5	1.91	1	0.38	15	5.74

**Table 2:** Summary of turnaround times between intermediate process phases

Time in minutes	Interval 1: registration acquisition on HIS, n=263	Interval 2: acquisition validation on HIS, n=263	Intra-lab TAT, n=263
Average	8	25	80
Median	29	20	56
Standard deviation	31	28	44
Interquartile range	20-40	09-35	36-80

Analysis results

**Figure 1:**
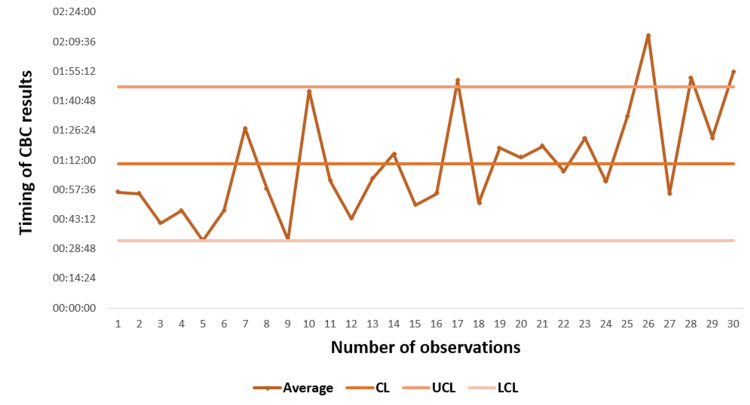
X-bar control chart of the means of the turnaround time for intra-laboratory blood count results obtained over one month of observation for 263 tests CBC: complete blood count; CL: central limit; UCL: upper control limit; LCL: lower control limit

Observation of the X-bar mean control chart of the TAT of the CBC results (Figure 1) revealed that the process was generally controlled. More than 93% of the intra-lab TATs were compliant. The process was out of control for two observations, points 26 and 30; it was above the UCL at these two points and took time to stabilize again.

**Figure 2:**
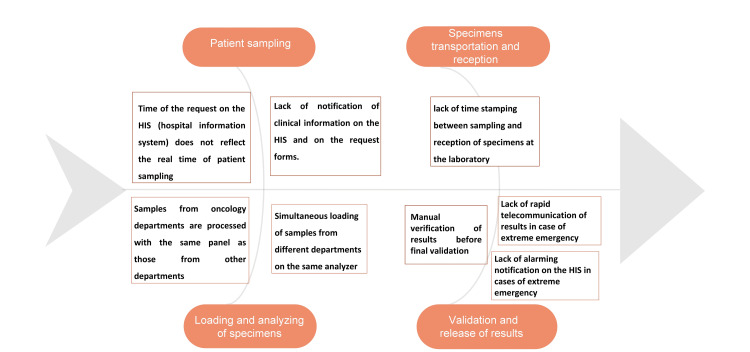
Ishikawa diagram to identify the root causes of failure of the entire process from the pre-analytical to the post-analytical phase

On the basis of an Ishikawa diagram (Figure 2), we revealed the following main causes: For the pre-analytical phase, we revealed a lack of time stamping of the time between the collection and the reception of the samples at the laboratory level. For the analytical phase, we revealed that samples from the oncology hospital were treated in the same panel as those of the other services. For the post-analytical phase, we revealed a lack of rapid communication of results or alarming notification on the HIS in cases of extreme urgency (low absolute neutrophil count (ANC) rate with evocative clinical signs).

Improvement results

In the pre-analytical phase, we could distinguish the request forms and also the labels of the samples of the oncology hospital by a particular color in order to alert the laboratory staff that these were samples to be treated in an emergency. It is also necessary to add any clinical sign suggestive of febrile neutropenia or COVID-19 infection, as well as information on empirical antibiotic therapy and the last round of chemotherapy. A section will be added to these request forms indicating the time of sample collection by the transport agent.

In the analytical phase (the creation of a specific chain for oncology department samples), it would be important to time-stamp the reception of the samples by the laboratory by loading them in specific boxes for oncology patients. These boxes will be labeled and differentiated by the same color as the request forms, which will make it easier for the laboratory technician to treat them as quickly as possible. The samples in question will be loaded and treated by the analyzer separately from those of other departments. Therefore, the treatment of the samples will be established according to a new, rapid, and exclusive panel.

In the post-analytical phase, it would be important to establish rapid and effective telecommunications between the hematology laboratory and the clinicians of the oncology hospital or to notify an alarm on the HIS for vital situations, which would make it possible to inform the treating clinician on site in real time. We could also propose that the calculation of the TAT be carried out automatically on the HIS, which would make it possible to notify the biologist of out-of-time analyses during biological validation stages. It is also possible to provide training of medical personnel by the laboratory biologist because this role is his responsibility according to the International Organization for Standardization (ISO) 15189 [20].

Control results: our objectives and estimations

First, we estimate that turnaround time will be improved after the implementation of the proposed flowchart measures. The overall TAT will be defined as the time from specimen collection to receipt in the laboratory, from receipt to registration of the request, from registration to completion of the analysis, and from analysis to authorization of the release of the validated results to the system. In fact, in order to respect the medical emergency that is the examination of the ANC count, the laboratory has set itself a time limit (global TAT) of a maximum of two hours and 30 minutes to give its result.

Considering that the oncology hospital is detached from the central laboratory of UHC, the time from sampling to the transportation of the samples to the laboratory will be a maximum of two hours. The average intra-laboratory TAT would be reduced from 80 minutes to 30 minutes: 15 minutes between the reception, registration, loading, and processing of samples on the analyzer and 15 minutes between result verification and releasing of validated results to the Laboratory Information System (LIS).

Second, mean values out of control of the intra-laboratory TAT of the CBC will be followed using another mean control chart. Third, we could even control the variations in the overall TAT process with another mean control chart. Fourth, knowledge of the TAT could allow clinicians to establish goals that are consistent and compatible with the clinical needs of their patients as well as those of the hematology laboratory. Fifth, our approach will have a contributing impact on the control and improvement of the quality of the entire analysis process, which is strongly correlated with the increase in patient satisfaction.

**Figure 3:**
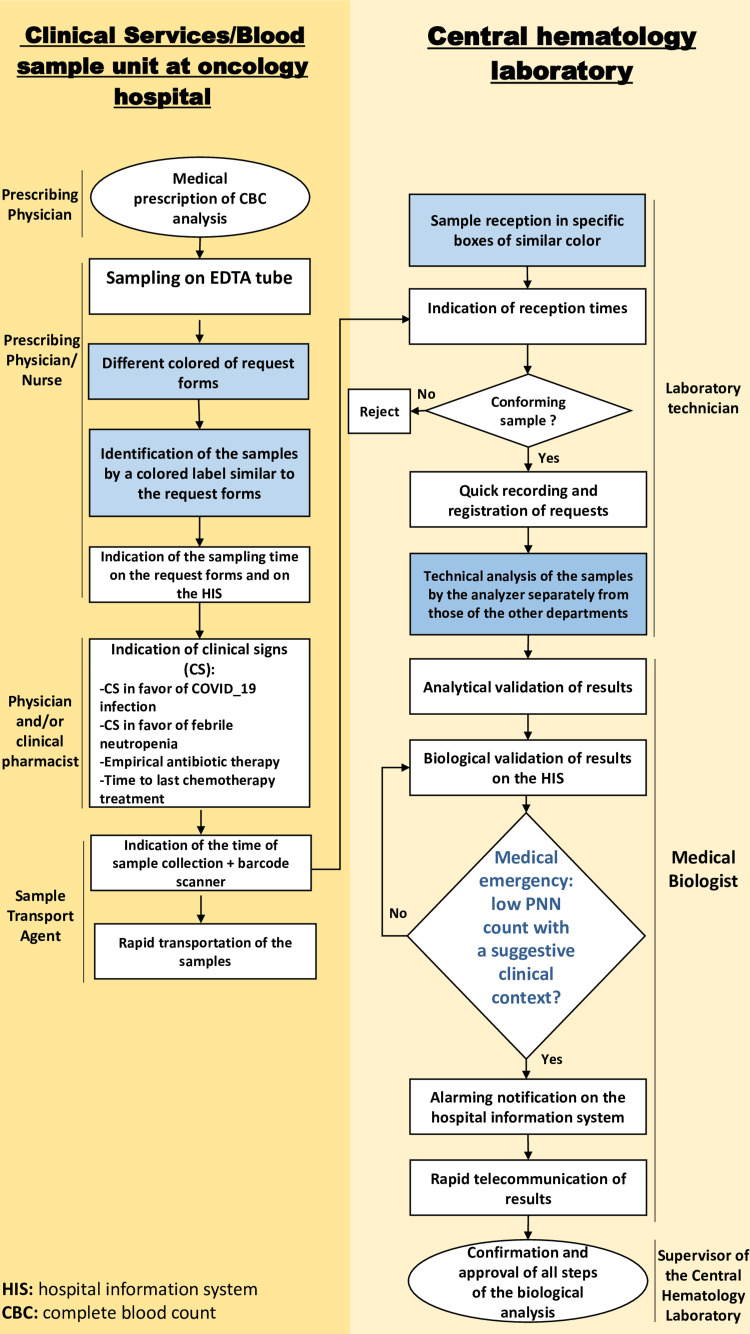
Suggested improvement flowchart

The suggested improvement flowchart is given in Figure 3.

## Discussion

The decision to urgently grant a chemotherapy session or even a possible transfusion is highly dependent on the results of the blood count and mainly on the ANC and platelets. The results of this study are compared with TAT studies in emergency departments. Our results showed that most samples were processed in the laboratory within 90 minutes and the median TAT-intra laboratory was 56 minutes (IQR: 36-80 minutes). These results are similar to the studies of Mutema et al. [13] and Mahdaviazad et al. [21]. In contrast, Winkelman et al. stated that this median time was 35 minutes for urgent hospital tests [22]. On the other hand, a generalized Chinese survey showed that the median intra-laboratory TAT for white blood cells and platelets was 44.7 minutes and 80 minutes, respectively [23].

The definition of TAT often varies between practitioners. The overall TAT is categorized into pre-analytical, analytical, and post-analytical stages [24]. In our study, the time taken into consideration is the TAT intra-laboratory, which is the time between the registration of the samples and the release of the results on the HIS that the laboratory could control. Generally, short TATs are necessary; the sample must be transported to the laboratory as soon as possible. A better CBC is one that is ideally performed between 30 minutes and two hours but does not exceed four hours given the collection, transportation, and analysis [25,26].

The observation of the X-bar average control chart revealed that the process is generally controlled. More than 93% of the intra-lab TATs were in compliance. The process was out of control only for two observations, points 26 and 30, which were above the UCL and took time to stabilize again. Overall, according to our study, the current intra-lab TAT is biologically acceptable. However, given the urgency of the cancer cases including neutropenia and the COVID-19 context, a number of procedures mentioned above (improved phase) that require rigorous organization will be put in place once our suggested model is accepted and adopted by all practitioners.

Study limitations

The pre-analytical phase is one of the most important steps in the biological cycle from the blood sampling to the return of the results. It constitutes a real informative value of the biological result. The first part of the pre-analytical phase is the blood sampling. According to the ISO 15189 guidelines [20], the biological sample must be accompanied by a request form that must contain the information necessary to identify the patient and the authorized prescriber, the relevant clinical data relating to the patient, and also the date and time of collection. However, the sample collection time is not communicated between the two departments (clinical service and laboratory).

There are two reasons for this anomaly. At the oncology hospital, the sample is usually taken in the dedicated sampling unit the day before the chemotherapy session. This is the reason why we could not extract the time of the sampling from the HIS. In another situation, it is done directly at the patient's bed when the patient is hospitalized and developing a post-chemotherapy complication. In this case, the time of the request does not really reflect the time of the sampling.

This study is also limited by the lack of an objective follow-up process for the previously proposed actions. Modeling of the proposed actions will aim to reduce the intra-laboratory time from 80 minutes to 30 minutes +/- five minutes and will also define the global TAT in two hours and 30 minutes. The reduction of these times will have a significant impact on the management of cancer patients undergoing chemotherapy, especially if they present signs suggestive of febrile neutropenia and/or infection by the coronavirus. A pilot study in this setting will be the focus of a future study.

## Conclusions

On the basis of available data, intra-laboratory TAT of CBC results of cancer patients was within a biologically acceptable range. Our work is certainly limited by the phases outside the control of the laboratory, but it should, however, initiate a continuous quality project, particularly an integration of the notifications in the HIS and a relevant communication between the two clinical-biological structures. The evaluation of the impact of the proposed improvement approach will be the subject of another study.

## Figures and Tables

**Figure 4:**
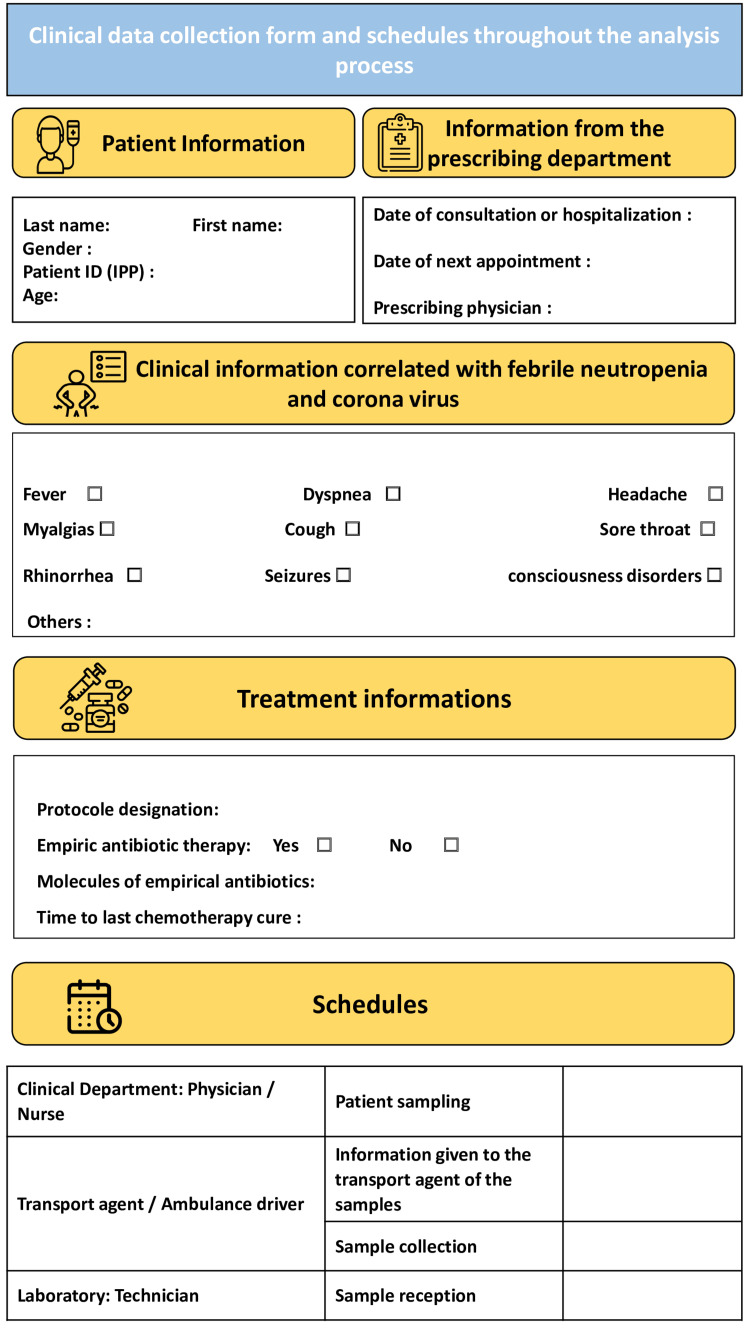
New request form for the oncology hospital

## References

[1] Hus I, Salomon-Perzyński A, Tomasiewicz K, Robak T (2021). The management of hematologic malignancies during the COVID-19 pandemic. Expert Opin Pharmacother.

[2] Lau SK, Luk HK, Wong AC (2020). Possible bat origin of severe acute respiratory syndrome coronavirus 2. Emerg Infect Dis.

[3] Addeo A, Friedlaender A (2020). Cancer and COVID-19: unmasking their ties. Cancer Treat Rev.

[4] Rais G, Amaoui B, Lahlou L, Ouazni M, Fares S (2020). Spécificités de la reorganization de la prise en charge des patients cancéreux au cours de la pandémie COVID-19 dans un centre régional d´oncologie au Maroc [Article in French]. Pan Afr Med J.

[5] Yu J, Ouyang W, Chua ML, Xie C (2020). SARS-CoV-2 transmission in patients with cancer at a tertiary care hospital in Wuhan, China. JAMA Oncol.

[6] Crawford J, Dale DC, Lyman GH (2004). Chemotherapy-induced neutropenia: risks, consequences, and new directions for its management. Cancer.

[7] Aapro M, Lyman GH, Bokemeyer C (2021). Supportive care in patients with cancer during the COVID-19 pandemic. ESMO Open.

[8] Cooksley T, Font C, Scotte F, Escalante C, Johnson L, Anderson R, Rapoport B (2021). Emerging challenges in the evaluation of fever in cancer patients at risk of febrile neutropenia in the era of COVID-19: a MASCC position paper. Support Care Cancer.

[9] Morjaria S, Zhang A, Kaltsas Md A (2020). The effect of neutropenia and filgrastim (G-CSF) in cancer patients with COVID-19 infection. medRxiv.

[10] (2022). COVID-19 rapid guideline: delivery of systemic anticancer treatments. https://www.nice.org.uk/guidance/ng161.

[11] Inal TC, Goruroglu Ozturk O, Kibar F, Cetiner S, Matyar S, Daglioglu G, Yaman A (2018). Lean six sigma methodologies improve clinical laboratory efficiency and reduce turnaround times. J Clin Lab Anal.

[12] Imoh LC, Mutale M, Parker CT, Erasmus RT, Zemlin AE (2016). Laboratory-based clinical audit as a tool for continual improvement: an example from CSF chemistry turnaround time audit in a South-African teaching hospital. Biochem Med (Zagreb).

[13] Mutema L, Chapanduka Z, Musaigwa F, Mashigo N (2021). In-depth investigation of turn-around time of full blood count tests requested from a clinical haematology outpatient department in Cape Town, South Africa. Afr J Lab Med.

[14] Bhat S, Gijo EV, Jnanesh NA (2014). Application of Lean Six Sigma methodology in the registration process of a hospital. Int J Product Perform Manag.

[15] Samanta AK, Varaprasad G, Gurumurthy A (2022). Reducing the turnaround time of laboratory samples by using Lean Six Sigma methodology in a tertiary-care hospital in India. 2nd Indian International Conference on Industrial Engineering and Operations Management.

[16] Umut B, Sarvari PA (2016). Applying lean tools in the clinical laboratory to reduce turnaround time for blood test results. Int J Adv Sci Eng Technol.

[17] (2011). Six Sigma Projects and Personal Experiences. Projects and Personal Experiences. BoD-Books on Demand.

[18] Nevalainen D, Berte L, Kraft C, Leigh E, Picaso L, Morgan T (2000). Evaluating laboratory performance on quality indicators with the six sigma scale. Arch Pathol Lab Med.

[19] (1995). Application of statistics. Control charts. Part 1: Shewhart control charts by variables. https://standardstore.afnor.org/en-gb/standard/nf-x060311/application-of-statistics-control-charts-part-1-shewhart-control-charts-by-/fa041150/14236.

[20] (2023). ISO 15189: 2022 quality assurance for medical laboratories!. ISO 15189.

[21] Mahdaviazad H, Javidialesaadi F, Hosseinzadeh M, Masoompour SM (2016). Turnaround times for hematology and chemistry tests in the emergency department: experience of a teaching hospital in Iran. Shiraz E Med J.

[22] Winkelman JW, Tanasijevic MJ, Wybenga DR, Otten J (1997). How fast is fast enough for clinical laboratory turnaround time? Measurement of the interval between result entry and inquiries for reports. Am J Clin Pathol.

[23] Fei Y, Zeng R, Wang W, He F, Zhong K, Wang Z (2015). National survey on intra-laboratory turnaround time for some most common routine and stat laboratory analyses in 479 laboratories in China. Biochem Med (Zagreb).

[24] Schimke I (2009). Quality and timeliness in medical laboratory testing. Anal Bioanal Chem.

[25] Harrison P, Mackie I, Mumford A, Briggs C, Liesner R, Winter M, Machin S (2011). Guidelines for the laboratory investigation of heritable disorders of platelet function. Br J Haematol.

[26] Bolodeoku J, Ogbeiwi O, Kuti MA, Adebisi SA (2017). Laboratory tests turnaround time in outpatient and emergency patients in Nigeria: results of a physician survey on point of care testing. Int J Med Res Health Sci.

